# Development of a Novel Micro Photoionization Detector for Rapid Volatile Organic Compounds Measurement

**DOI:** 10.1155/2018/5651315

**Published:** 2018-09-05

**Authors:** Qi Zhou, Sixiang Zhang, Xu Zhang, Xu Ma, Wei Zhou

**Affiliations:** ^1^School of Mechanical Engineering, Hebei University of Technology, Tianjin 300130, China; ^2^School of Mechanical Engineering, Tianjin University of Technology and Education, Tianjin 300130, China; ^3^School of Electrical and Electronic Engineering, Tianjin University of Technology, Tianjin 300130, China

## Abstract

The simulation of the gas flow field and electrostatic field in the photoionization detector by COMSOL was conducted based on principle investigation in the present study. Under the guidance of simulation results, structural optimization was carried out to significantly reduce the dead volume of the ionization chamber, and finally, the relationship between offset voltage and collection efficiency was obtained which led to a remarkable increase in the collection efficiency of charged ions in the photoionization detector. Then an ionization chamber with low interference and fast response was developed. Then experiment was performed with toluene as a VOCs gas under the condition of optimal gas flow rate of 50 ml, UV lamp ionization energy of 10.86 eV. The results showed that the ion collection efficiency reached 91% at a bias voltage of 150 V. Moreover, a preferred linearity of 99.99% was obtained, and a ppb level of LOD can be achieved. The determination results well-fitted the relationship between offset voltage and the response value obtained in the simulation.

## 1. Introduction

Volatile organic compounds (VOCs) is the general term of volatile organicity even at room temperature, which not only can influence our daily life but also can threaten human being's health [1]. By comparing the performance of different detectors, finding the appropriate VOCs detector, and comparing the working principle and performance characteristics of different detectors, the characteristics of different detectors are introduced in Table 1.

By comparing the performance of different detectors, it can be concluded that different types of detectors have different types of gases. The difference between the types of detectable gases is called the selectivity of the detector. The gas detectors with strong selectivity are limited by their own characteristics and are difficult to be widely used. Therefore, the detector used for VOCs detection should be a universal detector, the detection accuracy should be high, and the volume should be small, which is conducive to system integration and miniaturization design. In the detectors described above, the detection accuracy of FID is very high, but FID requires hydrogen as the combustion gas. The application range of ECD and ion migration spectrum detector is narrow, and it is not suitable for use as a VOCs detector. TCD has strong universality, but its low detection accuracy cannot achieve trace detection. Considering the characteristics of the above detectors, the photoionization detector is used as the terminal detector of the VOCs detection system [5].

In the early stage of the study, the UV light source and the ionization chamber were in the same space, which limits the sensitivity and accuracy of the detector. Later, as the research progressed, the scientists separated the two and made the detector more accurate. In practical work, the sensitivity of the photoionization detector is greatly influenced by the performance of the ionization chamber. After the introduction of the commercialized PID by HNU in 1976, companies such as RAE and Ion Science also introduced the PID detection devices [6]. However, these devices are complex in structure, lack scale production, are not competitive in the market, and cannot meet the requirements of trace detection. Recently, gas pollution is ubiquitous, and the research on PID is at a high level at home and abroad. The existing sensors still have the characteristics of large size, large power consumption, low efficiency, and high cost, and cannot meet the requirements for efficient portable detection [7].

The photoionization detector is a versatile, selective detector that responds to most organic compounds and acts as a nondestructive detector. This detector can be combined well with mass spectrometers or infrared detectors [8]. In practical work, the sensitivity of the photoionization detector was mainly determined by ionization chamber performance [9], and the detection level of PID was generally determined by the quantity of electron flow produced in the ionization chamber. Therefore, the design of the ionization chamber should be the key to PID design.

We have developed a microfluidic PID that can be used in a GC (*μ*GC) system for rapid and highly sensitive VOCs detection. The photoionization detector has been integrated into the GC (*μ*GC) system [10].

## 2. Method

**Figure 1:**
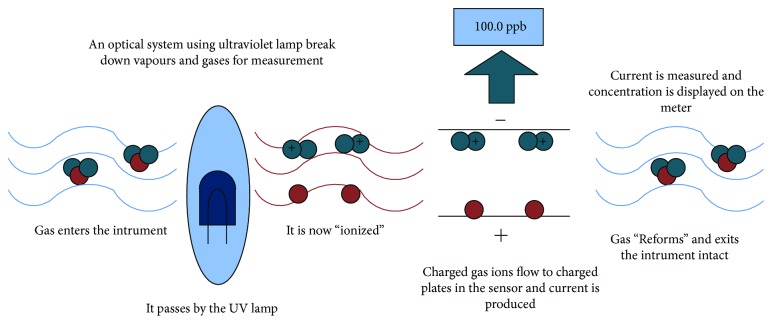
The working principle of PID.

As shown in Figure 1, the main reason for PID is that the charged ions are generated by the ultraviolet lamp through ionization of the gas between the electrodes. The main principle of PID is that the gas between the electrodes is ionized by ultraviolet light excited by the UV lamp to produce charged ions which can form a current when driven by a high voltage electric field. The current value is proportional to the concentration of charged ions generated between the electrodes. In combination with the separation of the chromatographic column, the qualitative or quantitative detection of the sample compound can be achieved based on the change in the current intensity between the electrodes over time [11].

The photoionization current was defined as the quantity of an ion pair generated by ionization per unit time and can be showed as

(1) $$\begin{array}{c} \frac{{dN}_{i}}{dt}=2\sigma i\phi \left[1-e^{-\sigma_{i}N\left(t\right)l}\right], \end{array}$$

with *N*_*i*_ denoting the quantity of the ion pair, *σ*_*i*_ denoting the absorption coefficient of photoionization, *ϕ* denoting the quantity of photons injected into the ionization chamber per unit time, *σ*_0_ denoting the absorption coefficient caused by other reasons, *σ*_*t*_ = *σ*_*i*_ + *σ*_0_ was the total absorption coefficient, *N*(*t*) was the amount of gas molecule to be determined, namely, the gas concentration to be determined, and *l* denoting the optical path length.

It can be seen from (1) that the quantity of the ion pair produced in the ionization chamber by excitation of gas molecules per unit time *dN*_*i*_/*dt* exhibited exponential relationship with VOCs concentration in the sample; (1) can be converted to

(2) $$\begin{array}{c} \frac{{dN}_{i}}{dt}=2\sigma_{i}{\varphi \sigma }_{t}N\left(t\right)\cdot l, \end{array}$$

within *σ*_*i*_*N*(*t*)*l* ≤ 1.

Then *dN*_*i*_/*dt* was in proportion to *N*(*t*), namely, as the value of *l*, the optical path of ultraviolet light, in the ionization chamber was short enough and the concentration of gas to be determined was relatively low, then the rough linear relationship of ionization current with gas concentration in the sample and *ϕ*, the quantity of photons injected into the ionization chamber per unit time, can be obtained [12]. Therefore, both the gas concentration of the sample and intensity of UV light may affect the strength of ionization current; hence, the ionization current will increase with both the value of gas concentration and UV light intensity.

In 1982, Freedman proposed the use of photoionization detector for gas detection, then the actual ionization current (*i*) was written as

(3) $$\begin{array}{c} i=I_{0}F\eta \sigma NL\left[AB\right], \end{array}$$

with *I*_0_ as the intensity of light radiation, *F* as the Faraday constant, *N* as the Avogadro constant, *η* as the absorption cross section of components, *σ* as the ionization efficiency of excited-state molecule, *L* as the thickness of light absorption layer, and [*AB*] as the gas component concentration to be determined [13].

In the parameters mentioned above, the values of *I*_0_ and *L* were associated with a PID structure, both values would be constant with a certain PID structure, the R value, namely, mole-basis response, was only determined by *η* and *σ*, and the relationship can be written as *R* = *i*/[*AB*] = *Kησ*. Among them, the product of *η* and *σ* was defined as the photoionization section, which was highly dependent on the ionization potential (*IP*) of gas molecules; hence, (3) can be written as

(4) $$\begin{array}{c} i=I_{0}FNL\left[AB\right]\left(IP\right), \end{array}$$

in which (*IP*) was a key factor that may affect the ionization response of PID.

According to the principle of photoionization, there are three main factors that affect the performance of photoionization: background noise, ionization chamber volume, and electron and ion collection efficiency. Among these three factors, background noise is impossible to avoid, which can cause the detection limit and baseline drift. So, minimizing the impact of background noise is the key element of optimization. The background noise is caused by the interference between the collector plates, which can be solved by optimizing the structure. Chamber volume of detection limit and the response speed is an important factor to ensure the volume as small as possible, so that it can get a lower detection limit and faster response speed and get a larger linear response area. Meanwhile, the detection limit and response speed can be improved by increasing the collection efficiency of electron and ion. The factors mentioned above can be improved by enhancing the sealing performance of the overall structure, reducing the ionization chamber volume, and increasing the bias voltage. According to the above analysis, we design the PID detector.

## 3. Description of the Model Equations

### 3.1. The Relationship between Electrode Offset Voltage and the Collection Intensity of Ionization Gas

The collection efficiency of the collecting electrode is of great significance to the overall sensitivity and stability of the detector. The finite element method was used to analyze the electrostatic field model to simulate the electrostatic field distribution in PID. In addition, based on the electrostatic field model, the particle tracking model in the software is further used to study the effect of the collecting electrode size on the collection efficiency of the charged particle, by which providing guidance for the structure design [14].

For the electric field distribution within PID was actually an electrostatic field issue, thus the Poisson equation shown in (5) can be used for simulation

(5) $$\begin{array}{c} \nabla^{2}\varphi =\frac{-\rho }{\varepsilon }, \end{array}$$

in which, *φ* was the electric potential, *ρ* was intensity of free charge, and *ε* was the dielectric constant of electrolyte. For no free charge distribution in the solution space, hence *ρ* = 0, then the Poisson equation can be further simplified as a Laplace equation shown in

(6) $$\begin{array}{c} \nabla^{2}=0. \end{array}$$

The finite element method electrostatic field model was used to simulate the simplified model of electrostatic field distribution in PID [15]. Based on the proposed electrostatic field model, the particle trajectory model was used to simulate the trajectories of charged ions in the PID to study the effect of collecting electrode size on the collection efficiency of charged particles.

**Figure 2:**
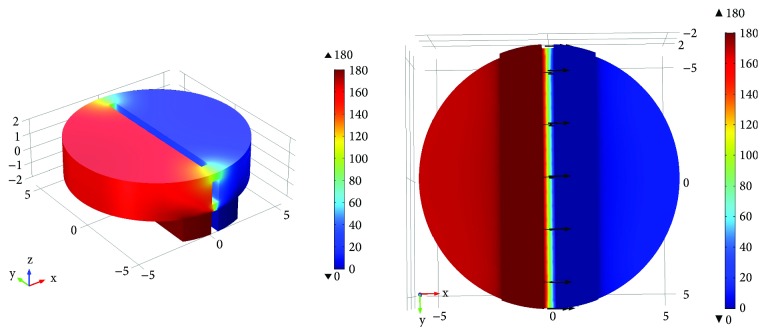
The distribution of collecting electrode potential and electric field lines.

As shown in Figure 2, the potential gradually decreases from the polarizing electrode to the collecting electrode. The electric potential line was convex to the collecting electrode. Between the biasing electrode and the collecting electrode, the electric field line is emitted from the top of the biasing electrode and converges on the inner surface of the collecting electrode. Which revealed that, with the effect of electric field force towards the collecting electrode, the positively charged particles formed on top of the polarization electrode would move towards the inner surface of the collecting electrode, while the negatively charged particles move towards the collecting electrode.

**Figure 3:**
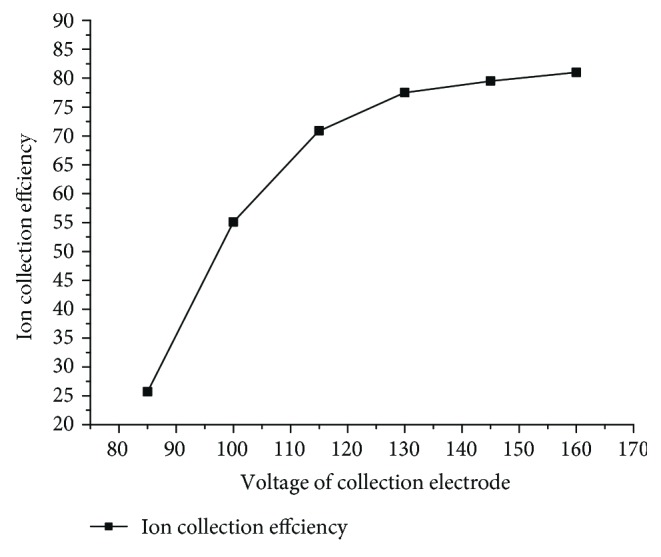
Effect of plate voltage of the collecting electrode on the charged ion collection efficiency.

As shown in Figure 3, most positively charged particles formed on top of the polarization electrode may rapidly move to the inner surface of the collecting electrode by the force of the electric field. Formula (7) can be employed to demonstrate the particle collection efficiency of PID, where the ratios of quantity of particles captured by the collecting electrode to the total quantity of particles emitted were employed to represent the particle collection efficiency of PID.

Through simulation, the collection voltage of the bias electrode under designed conditions can be obtained; meanwhile, the particle collection efficiency of PID can be obtained as

(7) $$\begin{array}{c} f\left(x\right)=0.0001492x^{3}-0.06939x^{2}+10.82x-485. \end{array}$$

### 3.2. The Relationship between the Length and Plate Distance of Collecting Electrodes and Ionization Gas Collection Efficiency

Given the even distribution of the electric field, then the period *t* for ions moved from one electrode to another can be deduced by the following equation:

(8) $$\begin{array}{c} t=\sqrt{\frac{2m}{eV}}\cdot L, \end{array}$$

in which *m* and *e* were the mass and charge of the ions, respectively, *V* was the offset voltage, and *L* was the polar plate distance. From which, for constant gas composition, the plate distance *L* may directly affect the sensor response speed. However, if the distance between the plates is too small, the gas cannot enter between the plates at a high gas flow rate so the ultraviolet light cannot be received for sufficient ionization. Therefore, under these two parameters, the design of the plate with the length of 7~11 mm guarantees the response speed of the sensor and the complete ionization of the gas after entering the plate [16].

Another parameter that can greatly affect the response speed was the polar plate distance of the collecting electrode, which can be obtained by the Gauss Law as follows:

(9) $$\begin{array}{c} \int \vec{D}\cdot d\vec{S}=\sum q=\int \rho \left(s\right)\cdot d\vec{S}. \end{array}$$

The *ρ*(*s*) in (9) was the surface density of charges, and the following equation can be obtained in the direction of the electric field when rectangular and parallel plates were employed:

(10) $$\begin{array}{c} \vec{D}=\rho =\frac{q}{S}, \end{array}$$

in which $\vec{D}$ was the electric displacement vector; the relationship between $\vec{D}$ and the electric field intensity $\vec{E}$ was as follows:

(11) $$\begin{array}{c} \vec{D}=\varepsilon \cdot \vec{E}. \end{array}$$

The electric field intensity of parallel plates was

(12) $$\begin{array}{c} \vec{E}=\frac{U}{d}. \end{array}$$

The relationship in the direction of the electric field can be derived by (11) and (12):

(13) $$\begin{array}{c} \vec{D}=\varepsilon \cdot \vec{E}=\varepsilon \cdot \frac{U}{d}. \end{array}$$

It can be seen from (13) that *D* was constant for a fixed electric field intensity and polar plate distance. Therefore, it can be revealed that the quantity of charge between the polar plates *q* was associated with the ion collection plate area *S*, and both the quantity of charges and ion current increased with the value of *S*, and hence, the sensitivity of PID would improve. Moreover, the ion collection efficiency of the ionization chamber can be obtained according to the Boag theory as

(14) $$\begin{array}{c} f={\left(1+\frac{\xi^{2}}{6}\right)}^{-1}, \end{array}$$

within *ξ* = *m* · *d*^2^ · *q*^0.5^/*V*, *d* was the polar plate distance, *V* was the voltage difference between the plates, *q* was the charge density of the air per unit time, and *m* was the correction factor for environmental effect. It can be concluded from (13) that the collection efficiency of the ionization chamber was associated with polar plate distance and polar voltage. For constant environment conditions, the higher ion collection efficiency can be obtained with smaller polar plate distance in the ionization chamber and more polar voltage difference [17].

Therefore, according to the gas flow rate requirements, an improved gas flow model is used to simulate the gas flow field. The channel width is 1 mm, the boundary condition of two carrier gas inlets on the top was set as constant with the gage pressure of 0.05 MPa, the outlet boundary condition was set as laminar flow with the value of 2*e* − 7 m^3^/s, and the inlet boundary condition was also set as laminar flow with the value of 2*e* – 8 m^3^/s [18].

According to the variability of liquid density with external conditions, the fluid can be classified into incompressible fluid and compressible fluid. The gas was usually regarded as compressible fluid, but when the gas flow rate is low, for example, when the Mach number Ma is less than 0.1 while the pressure change is small, the gas can be considered as an incompressible fluid. Therefore, with the gas flow rate of 50 ml/min, the Mach number of carrier gas can be obtained by

(15) $$\begin{array}{c} Ma=\frac{V}{a}, \end{array}$$

in which *V* was the fluid flow rate with the unit of m/s and *a* was the local sonic speed with the unit of m/s. By which, the Mach number of gas can be obtained as 1.95*e* − 4, which well-satisfied the conditions for incompressible gas flow.

The flow mode of fluid in the pipe can be divided into laminar flow and turbulent flow. As for laminar flow, the flow style of flow-cell within the fluid was stratified flow, and there was no mixture of radial flow and interlayer fluid; as for the turbulent flow, both radial flow and interlayer fluid within the fluid existed for the flow-cells, and the intense mixing among the flow-cells could be obtained. And the flow style of fluid can be distinguished by the Reynolds number (Re), in which the Reynolds number can be calculated by the following equation:

(16) Re=ρVLμ,

in which *ρ* was the fluid density with the unit of kg/m^3^, *V* was the fluid flow rate, with the unit of m/s, *L* was the characteristic length, with the unit of m, and *μ* was the dynamic viscosity, with the unit of Pa·s. The flow state of fluid can be determined by the comparison of the calculated Reynolds number with the critical Reynolds number. The flow state was laminar flow when Re ≤ Re_c_ and was turbulent flow when Re ≥ Re_c_. In the present study, for the calculated Reynolds number was Re = 17.49, which was far less than Re_c_, thus the corresponding flow state was viscous laminar flow [19].

In brief, the carrier gas flow in the circuit can be regarded as incompressible viscous flow, and the mathematical model can be proposed based on the Navier-Stokes equations:

(17) $$\begin{array}{c} \rho \left(\frac{\partial v}{\partial t}+v\nabla v\right)+\nabla P-\mu \Delta v=0, \end{array}$$

where *∇v* = 0.

In which the flow rate *v* and pressure *P* were unknown variables, *ρ* was fluid density, with the unit of kg/m^3^, and *μ* was dynamic viscosity, with the unit of Pa·s.

**Figure 4:**
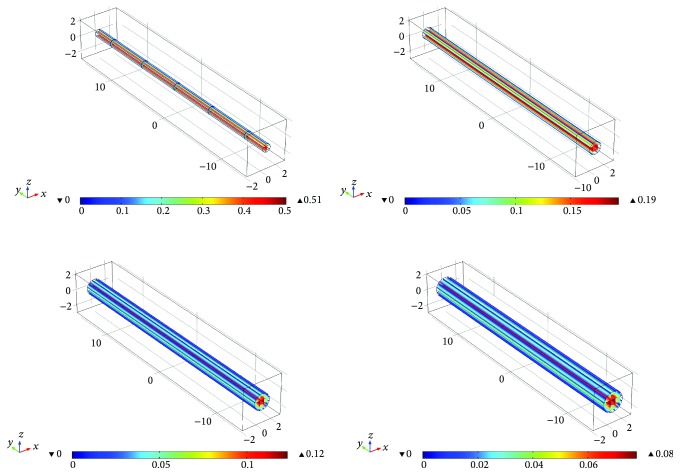
Simulation of the gas flow rate in the gas circuit.

The CFD module in COMSOL Multiphysics simulation software is used to simulate the gas flow field formed by the carrier gas flow in the gas path, and the results were shown in Figure 4.

Significant differences between the airflow pattern near the central area of the carrier gas inlet and the airflow pattern in other areas of the ionization chamber can be clearly observed. Among them, for an extremely small pipe diameter, a dead volume in an annular flow pattern may be formed due to a constant flow of 50 ml. And the presence of a relatively large dead volume can lead to a series of problems, such as widening of the chromatographic peaks, retention of the sample in the ionization chamber, and contamination of subsequent samples to be detected. Thus, it was necessary to reduce such areas during the design of the ionization chamber. Therefore, the diameter of the gas pipeline was designed to be 1.2 mm according to the diameter of the chromatographic column [20].

## 4. Experimental Section

### 4.1. System Characterization and Optimization

**Figure 5:**
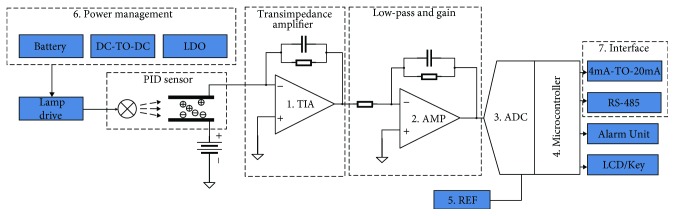
Schematic diagram of the miniature PID.

As shown in Figure 5, using borosilicate glass as the ionization chamber and electrode shell of the photoionization detector, the electrode is integrated into the etched channel using MEMS technology. By this way, the problem of the compact structure of the collector plates is solved. The ultraviolet lamp is mounted together with the electrode, and the sealing is guaranteed by the bonding process. Chromatographic columns are used as input and output channels, and weak signals are processed by the amplifier circuit.

**Figure 6:**
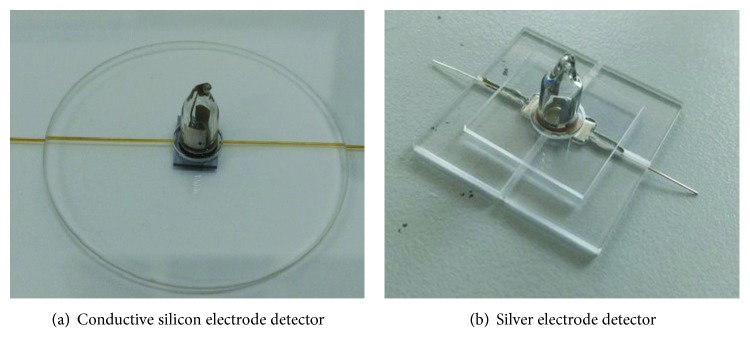
Miniature photoionization detector.

The miniature photoionization detector consists of a lamp, an upper sealing layer, an air chamber, an electrode, and a lower sealing layer. The lamp body uses the baseline line vacuum ultraviolet lamp (IP/N 043-257), the ionization energy is 10.6 eV, and the internal filling gas is krypton. The upper end sealing layer of the lamp is selected with a glass wafer of 0.5 mm, a 20 mm × 20 mm rectangle is cut through the laser etching method, and the opening is processed in the center area with a diameter of 8 mm. The lower sealing layer of the lamp also uses the glass wafer of BF33 with a thickness of 1 mm. Laser technology is used to etch the rectangular area of 30 mm × 30 mm, the air inlet, the air outlet, the electrode installation groove, and the conduit groove. The electrode with high conductivity is used as the collector of ion and electron, and the sealing layer is sealed by the anode bonding process. The inlet and outlet are connected by chromatographic column, and the two ends are sealed with UV curing adhesive. The opening and the ultraviolet lamp window of the upper sealing layer are sealed with UV curing adhesive. The miniature photoionization detector is shown in Figure 6.

The UV lamp is surrounded by two symmetrical copper sheets and activated by high voltage (100 kHz). This excitation method is similar to RF excitation and has the advantages of good reliability, uniform radiation intensity, and so on, but needs lower frequency. It can effectively reduce the electromagnetic interference to the rest of the circuit.

**Figure 7:**
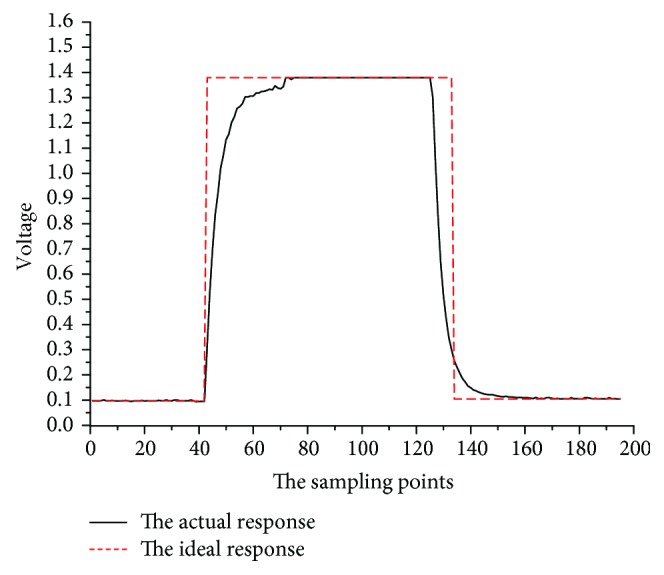
The sensor response process of the PID.

The determination process can be simplified as shown in Figure 7. The sample was introduced at time *t* = 60 and ended when it was stable. And the injection process is equivalent to the rectangular step shown in Figure 7:

(18) $$\begin{array}{c} x\left(t\right)=A\left[u\left(t\right)-u\left(t-t_{x}\right)\right],\;0\leq t<t_{y}, \end{array}$$

in which *A* was the steady value of the sensor responding to gas components. The sensor response value was associated with the gas flow rate and surrounding conditions. When the gas flow rate and surrounding conditions were constant, the sensor response value may only depend on the concentration and species of gas to be determined, and the steady value *A* may exhibit certain functional relationship with the gas concentration [21].

### 4.2. The Effect of Offset Voltage of the Paranoid Electrode on the Sensor Response

**Figure 8:**
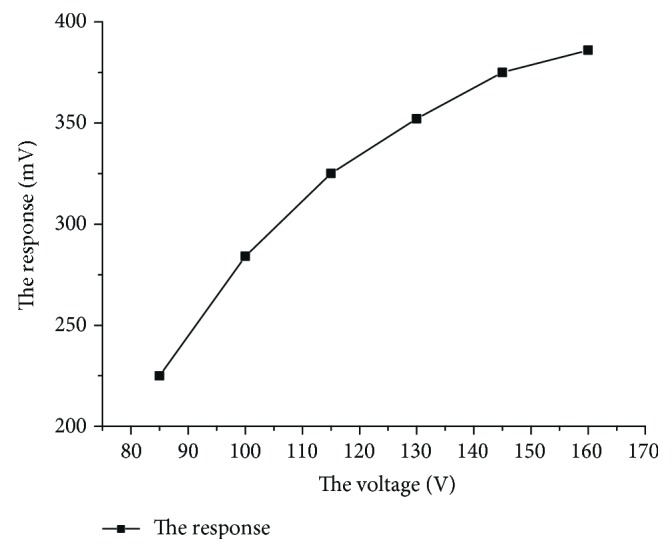
The effect of offset voltage on sensor response of the PID.

The suction flow rate of the diaphragm pump was maintained at 50 ml/min using the MFC (mass flow controller). The bias voltage is continuously increased from 85 V to 165 V for a certain voltage test point. Three sample injections (5 ppm toluene) were performed for each flow rate when both the system and background gases were steady, acquiring the response using a micro current amplifier, and the results were shown in Figure 8.

Polynomial fitting of the experimental data obtained was performed by the MATLAB software, where the *x*-axis indicated the offset voltage strength and the *y*-axis indicated the value of response, and then the following equation can be derived:

(19) $$\begin{array}{c} f\left(x\right)=0.0001402*x^{3}-0.07638*x^{2}+14.54x-539.2. \end{array}$$

As can be seen from Figure 8, the response value of toluene at 5 ppm gradually increases with the increase of the voltage, but the response value grows more slowly with the voltage. After reaching a certain voltage, the response voltage reaches a maximum value, and the response tends to stabilize as the voltage value continues to rise.

### 4.3. Linear Calibration of the Sensor

The gas with different toluene concentrations of 0.5 ppm, 1 ppm, 1.7 ppm, 2.5 ppm, and 5.1 ppm produced by a gas generator was employed in the present study, with nitrogen as the ambient gases, and 3 times of sample introduction for each concentration was conducted; the results were shown in Table 2.

**Figure 9:**
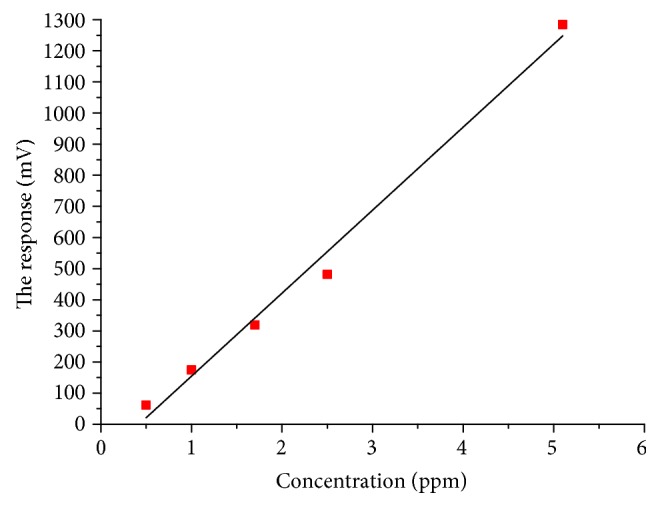
The effect of gas concentration on response value.

By the linear polynomial fitting of the experimental data shown in the table above, the following equation can be derived:

(20) $$\begin{array}{c} p=0.00394*C+0.2563, \end{array}$$

in which *p* was the response value, with the unit of mV, and *C* was the concentration, with the unit of ppm. The result was shown in Figure 9.

## 5. Conclusions

According to the analysis of experimental results, with toluene as the gas component to be determined, the relationship between the gas flow rate and response value of the PID sensor can well-satisfy the mechanisms of the photoionization sensor. The optimal working flow rate was in the range of 50 ml/min–70 ml/min. The finite element method was used to simulate the photoionization rate. The simulation results show that the structure of the photoionization detector is optimized. Ion collection efficiency reaches 91% with bias voltage of 150 V. Moreover, a preferable linearity of 99.99% of the photoionization detector was obtained and the LOD can reach ppb. The experimental results indicated that, in the range of 0 ppm–5 ppm, clear linear relationship of gas concentration with PID response value was obtained. The experiment proved that the micro photoionization detector can be widely applied to online detection of VOCs.

## Figures and Tables

**Table 1:** Performance comparison table of odor detectors.

Name	Abbr.	Applicable substance	Selectivity	Carrier gas	Linear range	LOD	Temperature upper limit
Thermal conductivity detector	TCD	All compounds	Nonselective	H_2_, He, N_2_	10^5^	10^−7^–10^−9^	450
Flame ionization detector	FID	Organic compound	No reaction to permanent gases or formic acid	H_2_, He, N_2_	5 × 10^6^–5 × 10^7^	10^−11^–10^−14^	450 [2]
Photoionization detector	PID	A compound below the maximum ionization energy	Most organic compounds	H_2_, He, N_2_, air	10^7^–10^8^	10^−13^	250 [3]
Electronic capture detector	ECD	Compounds containing oxygen or halogen	Good response to electronegative compounds	Ar, N_2_ + CH_4_	10^2^–10^4^	10^−13^–10^−14^	400
Ion migration spectrum detector	IMS	Organic matter and toxic substances [4]	Most organic compounds	He, N_2_	10^4^–10^5^	10^−10^	300

**Table 2:** Experimental results of concentration-response.

	0.5 ppm	1 ppm	1.7 ppm	2.5 ppm	5.1 ppm
	60	176	320	487	1288
Voltage value	62	173	317	478	1284
	60	175	319	479	1279
Average value	61	174.6	318.7	481.3	1283.7

Variance	1	1.52	1.53	4.93	4.51

## Data Availability

No data were used to support this study.
